# Limiting homologous recombination at stalled replication forks is essential for cell viability: *DNA2* to the rescue

**DOI:** 10.1007/s00294-020-01106-7

**Published:** 2020-09-09

**Authors:** Rowin Appanah, David Jones, Benoît Falquet, Ulrich Rass

**Affiliations:** 1 Genome Damage and Stability Centre, School of Life Sciences, University of Sussex, Falmer, Brighton, BN1 9RQ UK; 2 Friedrich Miescher Institute for Biomedical Research, 4058 Basel, Switzerland; 3 Faculty of Natural Sciences, University of Basel, 4056 Basel, Switzerland

**Keywords:** DNA replication stress, DNA replication fork, Homologous recombination, Chromosome stability, *DNA2* nuclease–helicase, Seckel syndrome

## Abstract

The disease-associated nuclease–helicase *DNA2* has been implicated in DNA end-resection during DNA double-strand break repair, Okazaki fragment processing, and the recovery of stalled DNA replication forks (RFs). Its role in Okazaki fragment processing has been proposed to explain why *DNA2* is indispensable for cell survival across organisms. Unexpectedly, we found that *DNA2* has an essential role in suppressing homologous recombination (HR)-dependent replication restart at stalled RFs. In the absence of *DNA2*-mediated RF recovery, excessive HR-restart of stalled RFs results in toxic levels of abortive recombination intermediates that lead to DNA damage-checkpoint activation and terminal cell-cycle arrest. While HR proteins protect and restart stalled RFs to promote faithful genome replication, these findings show how HR-dependent replication restart is actively constrained by *DNA2* to ensure cell survival. These new insights disambiguate the effects of *DNA2* dysfunction on cell survival, and provide a framework to rationalize the association of *DNA2* with cancer and the primordial dwarfism disorder Seckel syndrome based on its role in RF recovery.

## Introduction

In every cell cycle, cells face the enormous task of generating a faithful copy of their genome. Replisomes containing the Cdc45/Mcm2-7/GINS (CMG) replicative helicase unwind the parental chromosomes, forming DNA replication forks (RFs). At RFs, DNA polymerases ε and δ catalyse highly accurate leading- and lagging-strand DNA synthesis. The progression of RFs is routinely stalled by a range of obstacles including polymerase-blocking DNA lesions, DNA secondary structures, DNA-binding proteins, and DNA–RNA hybrids (Zeman and Cimprich 2014). Alterations in replication-origin firing caused by oncogene activation, depletion of replication factors, and low deoxyribonucleotide-triphosphate levels are further sources of replication stress that negatively impact RF progression. If stalled RFs are not properly dealt with, chromosomes remain partially unreplicated, jeopardizing chromosome disjunction at mitosis and the faithful transmission of the genome to daughter cells (Falquet and Rass 2019). Importantly, elevated replication stress has been linked to common fragile site expression, chromosome instability, and cancer (Gaillard et al. 2015; Glover et al. 2017).

In eukaryotes, DNA replication initiates at a multitude of replication origins, each giving rise to two RFs travelling in opposite directions along the parental chromosome. An inter-origin stretch of DNA is, therefore, replicated by a pair of converging RFs from adjacent origins (with the exception of DNA at the very tips of chromosomes), which provides protection against underreplication when individual RFs become blocked. In addition, dormant origins can activate in regions where DNA replication does not progress normally, providing another mechanism to make up for replication shortfalls upon RF-stalling or arrest. However, active and dormant origins are finite and cannot be set up de novo, while replication is ongoing (Siddiqui et al. 2013). Consequently, double-stalling events affecting a pair of converging RFs or single-stalling events in origin-poor regions of the genome cannot always be passively rescued by fork convergence to complete genome replication (Al Mamun et al. 2016). Mitigating the risk of local chromosome underreplication, cells have evolved mechanisms to protect, recover, and restart perturbed RFs, and homologous recombination (HR) proteins are intimately linked to these processes (Ait Saada et al. 2018).

## Homologous recombination-dependent replication restart

**Fig. 1:**
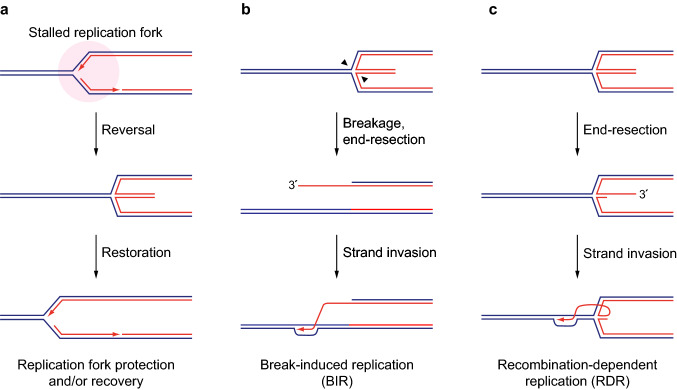
Processing and restart of stalled RFs. **a** When a stalled RF cannot easily resume DNA synthesis, it may backtrack into a reversed position, forming a four-way DNA junction. Reversed RFs have emerged as key intermediates of RF recovery, facilitating passive rescue by fork convergence or further processing to restore RF activity. **b** Breakage of stalled and reversed RFs can occur through attack by structure-specific nucleases (black arrowheads). The resulting single-ended DNA double-strand break undergoes DNA end-resection and Rad51-mediated strand invasion to initiate HR-dependent replication restart by BIR. **c** HR-dependent replication restart can also occur at unbroken reversed RFs. End-resection at the regressed branch produces the 3′-DNA overhang for Rad51-mediated strand-invasion, restarting replication along the RDR pathway

If stalled RFs remain replication-competent by retaining the replisome and their structural integrity, they may resume DNA synthesis once the replication impediment is resolved. However, stalled RFs quickly attract HR proteins (Nguyen et al. 2015), and more persistent RF-stalling renders replication completion more and more reliant on HR factors such as *RAD51* (Petermann et al. 2010). This is in large part due to protective functions of various HR factors at stalled RFs that are independent of signature recombination reactions such as homology search and DNA strand invasion mediated by the Rad51-single-stranded DNA (ssDNA) filament (Mason et al. 2019; Zellweger et al. 2015). Accordingly, human RAD51 has been shown to promote a process known as RF reversal, which entails the dissociation of the nascent leading and lagging strands from the parental template and their annealing with one another, essentially turning three-way RFs into four-way DNA junctions known as reversed RFs, chicken-foot structures, or Holliday junctions (Fig. 1a). Reversed RFs have emerged as key intermediates for the protection of stalled RFs and the resumption of DNA synthesis by a variety of mechanisms (Neelsen and Lopes 2015). RAD51 and its primary loader in mammalian cells, BRCA2, protect the DNA end exposed at reversed RFs from degradation, maintaining them in a configuration that allows subsequent restart and/or fusion with an approaching active RF (Berti et al. 2020; Schlacher et al. 2011). Similarly, fork protection by Rad51 and its loader Rad52 is indispensable for replication completion upon RF-blockage in yeast (Ait Saada et al. 2017; Pardo et al. 2020).

Long-term exposure to replication stress is associated with the breakage of stalled RFs (Petermann et al. 2010), at least in part due to the action of structure-specific nucleases (also known as Holliday-junction resolvases) (Rass 2013). Nucleolytic cleavage results in severance of one sister chromatid from the fork and the formation of a single-ended DNA double-strand break. DNA end-resection at the broken sister chromatid results in the formation of a 3′-overhang, which can serve as a substrate to prime renewed DNA synthesis by DNA polymerase δ in the context of a displacement loop (D-loop) upon Rad51-mediated strand invasion of the unbroken parental chromosome. This pathway, which has been studied extensively in the budding yeast *Saccharomyces cerevisiae* (Lydeard et al. 2007, 2010), is known as break-induced replication (BIR) (Kramara et al. 2018) (Fig. 1b). During BIR, D-loop migration and extensive DNA synthesis are uniquely dependent upon the conserved helicase *PIF1* (Saini et al. 2013; Wilson et al. 2013). Although the fate of the CMG replicative helicase during HR-dependent replication restart is not entirely clear, substituting CMG with Pif1 during D-loop DNA synthesis may solve the problem that the replicative helicase, once lost, cannot reload onto DNA in the S phase of the cell cycle.

A mechanism similar to BIR operates to restart stalled but unbroken RFs. Using the site-specific RF barrier provided by *Schizosaccharomyces pombe RTS1* (replication termination sequence 1), it has been demonstrated that persistently stalled RFs restart through recombination-dependent replication (RDR) (Ahn et al. 2005; Lambert et al. 2005). Stalled RFs are engaged by the double-stranded DNA (dsDNA) end-binding protein Ku, which suggests that RF reversal precedes RDR (Teixeira-Silva et al. 2017). Ku stabilizes the reversed-fork structure, but can subsequently be removed through nucleolytic cleavage mediated by the Mre11–Rad50–Nbs1 (MRN) complex and CtIP. MRN-CtIP action results in limited DNA end-resection, followed by more extensive resection by Exo1, which generates a 3′-overhang for intramolecular, Rad51-dependent strand invasion ahead of the site of fork reversal to restart replication (Fig. 1c). DNA synthesis within the resulting D-loop is dependent—as in the case of BIR—on Pif1 (Jalan et al. 2019).

## Homologous recombination-dependent replication: a salvage pathway with a cost to genetic stability

RF protection and restart by HR factors is vital for the completion of DNA replication, particularly under replication stress conditions. However, BIR and RDR are associated with a cost to genetic stability: BIR is characterized by high mutation frequencies (Deem et al. 2011) and the D-loop structures involved are intrinsically unstable. Thus, nascent DNA strands may undergo multiple strand eviction/invasion episodes, increasing the risk of ectopic recombination and formation of chromosome rearrangements (Costantino et al. 2014; Lambert et al. 2010; Mayle et al. 2015; Mizuno et al. 2013; Smith et al. 2007). While BIR/RDR can proceed for thousands of nucleotides, fusion with oncoming conventional RFs usually limits the extent of error-prone replication. In addition, the aforementioned mechanisms attenuating DNA end-resection at stalled and remodelled RFs antagonize the commitment to HR-dependent replication restart. For example, the initial protection by Ku from end-resection would favour a merger of a stalled RF with an approaching fork over RDR (Teixeira-Silva et al. 2017). Similarly, the resection nuclease Exo1 is constrained by the S-phase checkpoint (Tsang et al. 2014), while various regulators of RF remodelling and degradation have been identified in mammalian cells (Rickman and Smogorzewska 2019). Thus, DNA end-resection is a critical and highly controlled juncture for RF protection versus HR-dependent restart of stalled RFs. In budding yeast, protein sumoylation mediates the relocation of perturbed RFs to the nuclear pore, promoting Rad51-dependent replication restart and suggesting a spatial control mechanism governing commitment to HR-dependent replication restart (Nagai et al. 2008; Whalen et al. 2020). Quite clearly, cells carefully balance the need to restart persistently stalled RFs to avoid chromosome underreplication against the potential cost to genome stability that is associated with HR-dependent replication mechanisms.

## Anything in excess is a poison: Dna2 limits homologous recombination-dependent restart at stalled replication forks

Our recent work suggests that the Dna2 nuclease–helicase plays a critical role in limiting RDR at stalled RFs (Falquet et al. 2020). *DNA2* is essential across organisms and implicated in a variety of DNA metabolic processes including DNA double-strand break repair, checkpoint activation, Okazaki fragment processing, telomere homeostasis, centromeric DNA replication, and RF recovery (Zheng et al. 2020). In budding yeast, it has long been known that the lethality associated with loss of *DNA2* can be suppressed by deletion (or nuclear exclusion) of *PIF1* and/or DNA damage-checkpoint mediator *RAD9*. In the absence of Dna2, Pif1 and Rad9, therefore, mediate a toxic process. To explain this, it has been proposed that Pif1 stimulates strand-displacement DNA synthesis by DNA polymerase δ on the lagging strand, resulting in Okazaki fragments with extended 5′-flaps; if not cleaved by Dna2, these flaps are thought to associate with ssDNA-binding protein RPA, eliciting a Rad9-dependent DNA damage-checkpoint response, cell-cycle arrest, and ultimately cell death. These considerations have led to the concept that *DNA2* fulfils its essential function by policing the generation of long 5′-flaps during Okazaki fragment maturation (Budd et al. 2011). However, overt lagging-strand DNA replication defects associated with *DNA2* dysfunction have failed to transpire in yeast or human (Duxin et al. 2012; Kahli et al. 2019).

We found that—in the absence of *DNA2* and *PIF1*, or *DNA2* and *RAD9*—budding yeast cells fail to complete chromosome replication (Falquet et al. 2020), which is in keeping with Dna2′s documented role in stalled-RF recovery (Hu et al. 2012; Ölmezer et al. 2016; Thangavel et al. 2015). Under these conditions, survival became dependent upon Yen1, a Holliday-junction resolvase that is activated in anaphase (Blanco et al. 2014; Garcia-Luis et al. 2014; Ip et al. 2008), and which resolves persistent replication intermediates to facilitate viable chromosome segregation in *DNA2*-defective and *dna2*Δ cells (Falquet et al. 2020; Falquet and Rass 2017; Ölmezer et al. 2016). This showed that *dna2*Δ cells suffer from severe replication defects, and require failsafe resolution of underreplicated chromosomes by Yen1, and that these defects are fully independent of any action of Pif1. Accordingly, *dna2*Δ *pif1*Δ cells exhibit hypersensitivity upon exposure to exogenous replication stress. Next, we addressed whether the toxicity that is generated if Pif1 and Rad9 are present might be related to stalled RFs that are not properly processed when Dna2 is dysfunctional. Using hypomorphic *dna2* cells, which—in contrast to *dna2*Δ cells—can be grown in the presence *PIF1*, we determined that Pif1 exacerbates *dna2*-linked DNA replication problems. Thus, Pif1 proved responsible for an unscheduled, post-replicative checkpoint response in *dna2* cells that we have previously described (Ölmezer et al. 2016). This checkpoint response occurs immediately following passage through S phase under replication stress conditions, is distinct from Mec1–Ddc2/Mrc1/Rad53-mediated replication checkpoint signalling, and fully dependent upon DNA damage-checkpoint mediator Rad9. Consequently, Dna2 dysfunction in the presence of stalled RFs renders cells susceptible to cell-cycle arrest at the G2/M transition, which is enforced by the DNA damage checkpoint and elicited by the actions of Pif1. We surmised that RFs, which are not properly processed in the absence of *DNA2*, become susceptible to Pif1-mediated fork transitions, ultimately resulting in DNA damage-checkpoint activation and cell death. This provided an alternative to the Okazaki fragment processing model by explaining the essential nature of *DNA2* with its role in stalled-RF recovery (Falquet et al. 2020).

What might toxic fork transitions mediated by Pif1 consist of? While Pif1 is a versatile helicase with multiple functions in DNA replication and repair (Chistol and Walter 2014), we found that unrelated point mutations within a stress-responsive phosphorylation motif and Pif1’s PCNA-interacting PIP box suppressed unscheduled DNA damage-checkpoint activation in response to RF-stalling and cell death in hypomorphic *dna2* cells and *dna2*Δ cells, respectively (Falquet et al. 2020). Both of these Pif1 mutations converge on a specific Pif1 activity, blocking its ability to promote HR-coupled DNA synthesis (Buzovetsky et al. 2017; Vasianovich et al. 2014). From these observations, we concluded that Pif1/Rad9 toxicity is a direct consequence of inappropriate HR-dependent replication restart at RFs that are not properly processed by Dna2.

Given that HR-dependent replication restart provides a means to reboot DNA synthesis at perturbed RFs and complete DNA replication under stress conditions, why should this pathway be harmful in *DNA2*-defective cells? An important clue to answer this question came from a recent study investigating the types of aberrant DNA structures that accumulate upon depletion of Dna2 using electron microscopy (Rossi et al. 2018). In line with the previous findings in yeast and human cells (Hu et al. 2012; Thangavel et al. 2015), the authors found elevated levels of DNA four-way junctions, suggesting stalled RFs that escape processing by Dna2 accumulate in a reversed-fork configuration. Another intermediate, observed at even higher levels, consisted of dsDNA with one long branch of ssDNA that sometimes exceeded 10,000 nucleotides, representing a significant trigger for checkpoint activation upon coating with RPA. Since the Okazaki fragment processing machinery is biased against the formation of long DNA flaps, and extensive strand-displacement synthesis by DNA polymerases δ does not occur on the chromatinised lagging strand in vivo (Devbhandari et al. 2017; Garg et al. 2004; Kahli et al. 2019; Smith and Whitehouse 2012; Stodola and Burgers 2016), we suggest that these dsDNA/ssDNA intermediates are generated through RDR at stalled RFs by way of D-loop collapse. We propose a model where Dna2 counteracts RF reversal by degrading the nascent DNA strands at stalled RFs (Hu et al. 2012; Thangavel et al. 2015). Upon Dna2 dysfunction, reversed RFs accumulate and become available for HR-dependent replication restart. Consequently, there is excessive RDR (which can be suppressed by disrupting *PIF1*) and extensive D-loop DNA synthesis of thousands of nucleotides worth of DNA. Given the intrinsic instability of D-loops, nascent strands may disengage and remain permanently exposed as ssDNA. Accumulating levels of RPA-bound ssDNA will then activate the checkpoint (which can be suppressed by disrupting *RAD9*), causing a futile cell-cycle arrest in Dna2-deficient cells.

## Future questions and concluding remarks

**Fig. 2:**
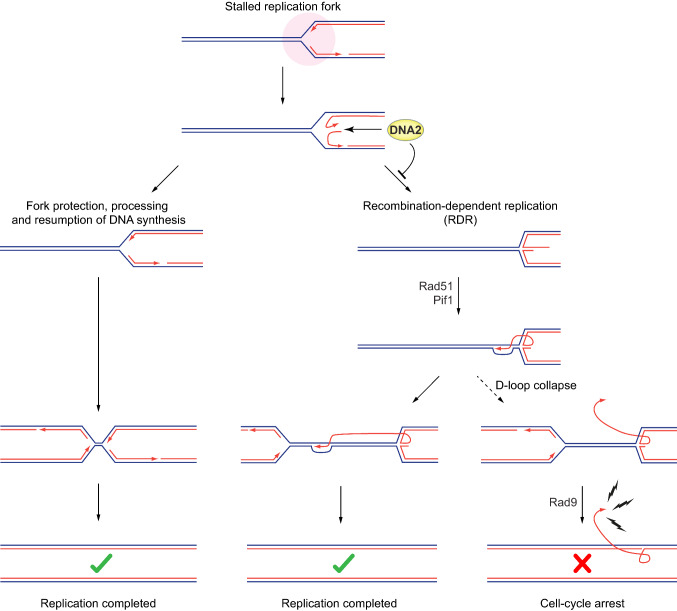
Dna2 is an essential gatekeeper to HR-dependent restart of stalled replication forks. By degrading dissociated nascent DNA at stalled RFs, Dna2 counteracts RF reversal, promoting the resumption of DNA synthesis and/or RF convergence to mediate complete genome replication. By processing stalled RFs, Dna2 also limits opportunities for RF restart by RDR, and this makes Dna2 indispensable for cell survival. While RDR provides an important salvage pathway for stalled RFs, DNA synthesis in the context of a displacement loop (D-loop) is unstable. In the absence of Dna2, excessive RDR results in an accumulation of unsustainably high levels of recombination by-products by way of D-loop collapse. Exposure of long ssDNA tracts then causes Rad9-dependent checkpoint activation and terminal cell-cycle arrest. For further details, see main text

As shown in the model presented in Fig. 2, we envisage a dual role that makes Dna2 essential: first, Dna2 promotes RF recovery, thereby facilitating complete genome replication; second, Dna2 acts as gatekeeper to RDR, guarding cells against the lethal consequences of an excessive use of HR-dependent replication restart at stalled RFs. It remains unclear how the actions of Dna2 might integrate with other replication and repair factors at stalled RFs, limiting HR-dependent replication restart while allowing for it when required. Regulatory mechanisms could involve Dna2 phosphorylation (Chen et al. 2011; Hu et al. 2012), sumoylation (Ranjha et al. 2019), or protein inhibitors (Higgs et al. 2015). The exact processing reaction catalysed by Dna2 at stalled and/or reversed RFs also remains to be determined. Dna2 shows preferential binding of complex DNA structures including partially reversed RFs with a dissociated leading or lagging strand exposed as ssDNA (Park et al. 2020). Since Dna2 is capable of degrading ssDNA from the 5′- and 3′-ends (Cejka et al. 2010), its actions could, in principle, remove the dissociating nascent leading and lagging stands at stalled RFs as proposed previously (Hu et al. 2012), effectively pre-empting their annealing and formation of reversed RF structures. This is very different from the well-established role of Dna2 in DNA end-resection during DNA double-strand break repair by HR, where, alongside Exo1, Dna2 cooperates with the Sgs1 helicase (BLM in human) to unwind the DNA duplex and, stimulated by RPA, specifically degrades the 5′-terminated DNA strand (Cejka et al. 2010). Interestingly, in fission yeast, Exo1, but not Dna2-Rqh1 (the Sgs1 homologue), has been implicated in end-resection at reversed RFs to generate the 3′-overhand needed for RDR (Teixeira-Silva et al. 2017). Moreover, Pxd1 has been shown to associate with Dna2, suppressing the stimulatory effect that RPA has on Dna2 activity (Zhang et al. 2014). Dna2 activity thus appears malleable in a context-specific manner, but more work is required to determine whether Dna2 indiscriminately degrades nascent DNA strands at stalled RFs. While protection from replication stress depends chiefly on Dna2’s nuclease activity (Falquet et al. 2020), its helicase activity contributes to RF recovery (Ölmezer et al. 2016). This suggests that at least a subset of replication intermediates requires unwinding as well as DNA degradation by Dna2; this activity may target fully reversed RFs, for which Dna2 also shows preferential binding (Park et al. 2020).

In conclusion, cells are set up to protect stalled RFs, resume DNA synthesis where possible, and ultimately rescue more persistently stalled forks using HR-dependent replication restart. While RDR/BIR are associated with a cost to genetic stability, their use reflects the overriding necessity to ensure that chromosomes are fully replicated before cell division. Our work indicates that RDR by-products can lead to cell death by eliciting a checkpoint-mediated cell-cycle arrest, uncovering an additional danger linked to the promiscuous use of HR-dependent replication restart. *DNA2* averts this danger through the processing of stalled RFs, thereby limiting opportunities for RDR (Falquet et al. 2020). These findings provide a new rationale for the essential requirement of *DNA2* for cell viability, which consists of establishing the correct balance between different RF recovery and restart pathways. Based on our work, an unbalanced response to RF-stalling resulting in diminished cell proliferation during development (Klingseisen and Jackson 2011) provides a plausible explanation for causative *DNA2* mutations in patients with the primordial dwarfism disorder Seckel syndrome (Shaheen et al. 2014; Tarnauskaitė et al. 2019). In cancer, *DNA2* has been found overexpressed (Peng et al. 2012; Strauss et al. 2014), potentially providing a survival advantage under elevated replication stress, which is prevalent in cancer cells. In future, it will be important to further address the RDR gatekeeper role of *DNA2* in human cells.
